# Evaluation of tip and torque on virtual study models: a validation study

**DOI:** 10.1186/2196-1042-14-19

**Published:** 2013-07-26

**Authors:** Luis T Huanca Ghislanzoni, Megan Lineberger, Lucia HS Cevidanes, Andra Mapelli, Chiarella Sforza, James A McNamara

**Affiliations:** Dipartimento di Scienze Biomediche per la Salute, Università degli Studi di Milano, Milano, Italy; Orthodontic Program, University of Michigan, Ann Arbor, Michigan USA; Department of Orthodontics and Pediatric Dentistry, School of Dentistry, University of Michigan, Ann Arbor, Michigan USA; School of Medicine, University of Michigan, Ann Arbor, Michigan USA; Center for Human Growth and Development, University of Michigan, Ann Arbor, Michigan USA

## Abstract

**Background:**

The objectives of this study were to develop and validate a novel analysis protocol to measure linear and angular measurements of tip and torque of each tooth in the dental arches of virtual study models.

**Methods:**

Maxillary and mandibular dental casts of 25 subjects with a full permanent dentition were scanned using a three-dimensional model scanner. Sixty points per arch were digitized on each model, five points on each tooth. A custom analysis to measure linear distances and angles of tip and torque was developed using a new reference plane passing as a best-fit among all of the lingual gingival points, with the intermolar lingual distance set as the reference *X*-axis. The linear distances measured included buccal, lingual, and centroid transverse widths at the level of canines, premolars, and molars as well as arch depth and arch perimeter.

**Results:**

There was no systematic error associated with the methodology used. Intraclass correlation coefficient values were higher than 0.70 on every measure. The average random error in the maxilla was 1.5° ± 0.4° for torque, 1.8° ± 0.5° for tip, and 0.4 ± 0.2 mm for linear measurements. The average random error in the mandible was 1.2° ± 0.3° for torque, 2.0° ± 0.8° for tip, and 0.1 ± 0.1 mm for the linear measurements.

**Conclusions:**

A custom digital analysis protocol to measure traditional linear measurements as well as tip and torque angulation on virtual dental casts was presented. This validation study demonstrated that the digital analysis used in this study has adequate reproducibility, providing additional information and more accurate intra-arch measurements for clinical diagnosis and dentofacial research.

## Background

The analysis of dental casts is an essential step in orthodontic diagnosis and treatment planning. A number of systems for on-screen measurements of virtual three-dimensional (3D) study models have been proposed in the literature to replace the time-consuming traditional manual measurements on plaster casts [1–5]. 3D virtual casts are an appropriate and accurate reproduction of the dental arch morphology for both indirect scanning systems from plaster casts and direct intraoral scanner acquisitions [6]. Digital measurements have been shown to be as reliable as manual measurements with a caliper [1–4]. The digital dimension extends the diagnostic and research tools for both clinicians and researchers, allowing them to take measurements of angles of tip and torque, surfaces, and volumes [7].

As orthodontists, we are concerned about the position of each individual tooth within the dental arches, including the angulation of the teeth in the mesiodistal (tip) and faciolingual (torque) dimensions. Clinicians are continually faced with various tip and torque prescriptions of each commercially available bracket system and are often unable to determine the extent to which the teeth follow the movement designated by the prescription. 3D virtual casts allow the use of additional tools to measure tip and torque thus deepen the understanding of what happens to each tooth during treatment.

Through advances in manufacturing capabilities today, it is possible to build custom prescription brackets and aligners based on virtual setups of the dentition [8–12]. There have been attempts to measure intermolar and interincisal angles on plaster casts that have been trimmed, sectioned, and photocopied; however, accuracy is difficult to achieve using this approach [13]. For example, questions have arisen regarding the accuracy of the work of Andrews [14] on tip and torque measured with a protractor because a repeatability test was not reported in his original work. More recent studies [15–17] have repeated Andrews' work on different samples; however, their aim was to compare the findings concerning average tip and torque values rather than evaluating the accuracy of the methodology. Where reported, a fairly high range of variability (1.3° to 4.0°) was found [16].

Due to the irregular convexity of the facial surface of a tooth, it is difficult to measure the inclination reliably with the methodology used in previous studies [15]. Early attempts have been made to create a more precise custom analysis that provides tip and torque data by digital acquisition of points through a magnetic field [18]. These data do not reflect how orthodontists define tip and torque because the studies described the inclination of the facial axis of the clinical crown (FACC) on the *X*- and *Y*-axes of a XYZ reference system. To measure the tip and the torque of each tooth requires a customized reference system.

The aims of the present study were to develop and validate a custom digital dental analysis to measure traditional linear measurements (e.g., transverse width, arch depth), as well as angular measurements of tip and torque of each tooth on virtual study models. Specifically, the validation of the analysis proposed in this study was performed to test its reproducibility as a diagnostic and research tool.

## Methods

### Subjects and methods

Sample size was determined on the basis of a pilot study [19]. In order to detect an effect size of 0.6 for the average tip and torque angles, with a desired power of 0.80 and an alpha of 0.05, the sample size should be at least 24 dental casts. Maxillary and mandibular dental casts of 25 subjects (13 males, 12 females; age range 12 to 18 years) with a full permanent dentition through the first molars, no dental anomalies or craniofacial syndromes, and no cast restorations or cuspal coverage were selected from a parent sample of 60 subjects. The second molars often were absent or erupting and therefore were excluded from the analysis. In total, 25 maxillary dental arches and 25 mandibular dental arches from the same subjects were available to test the validity of the virtual analysis of the dentition. The study protocol was approved by the local ethic committee and all the analyzed individuals gave their informed consent to the experiment.

The dental casts were scanned by way of the ESM/3ShapeTMR-700 three-dimensional model scanner (ESM Digital Solutions, Dublin, Ireland) and converted into .stl files. The VAM software (Vectra, Canfield Scientific, Fairfield, NJ, USA) was used to edit the files by placing 60 points per arch, according to the following protocol.

### Point digitization

**Figure 1:**
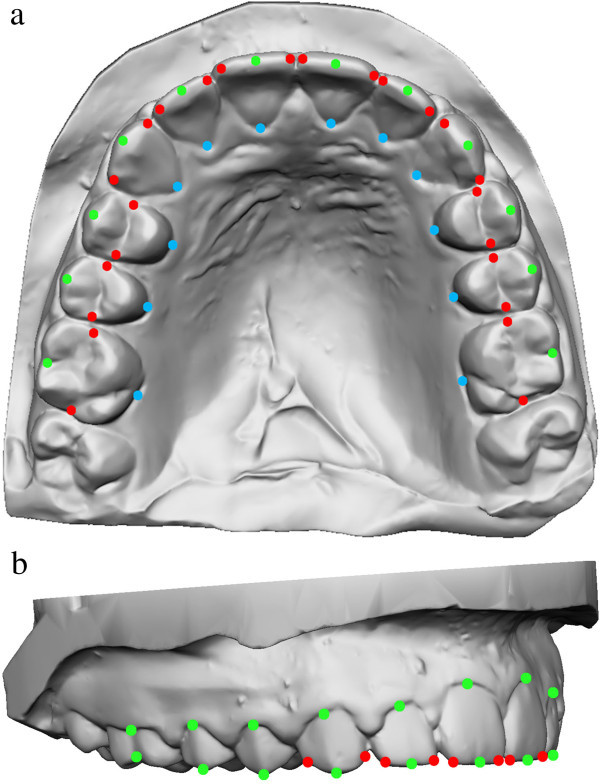
**Maxillary dental arch showing distribution and position of 60 landmarks from occlusal (a) and lateral (b) perspectives.**

The 60 points (Figure 1) were digitized according to the following guidelines:

-  Five points were taken for each tooth: the mesial and distal points of the occlusal surface, the gingival and occlusal limits of the buccal FACC [14], and the gingival limit of the lingual FACC (continuation of the buccal FACC on the lingual surface).
-  The most mesial and distal points of the occlusal surface of each tooth were digitized. The term occlusal surface is appropriate for molars and premolars, while for incisors, it is represented by the incisal edge and for the canines the canine ridges.
-  For incisors, canines, and premolars, the buccal and lingual FACCs were identified three-dimensionally as the lines passing through the most prominent portion of the buccal surfaces and their projection onto the lingual surfaces. For molars, the buccal and lingual FACCs corresponded to the dominant vertical grooves on the buccal and lingual surfaces of the crown, respectively. Gingival and occlusal limits of both the buccal and the gingival FACCs limit the lingual FACC and then were digitized.

After checking for the consistency of point order [20], the operator exported the point coordinates (XYZ) as a .txt file. Digitization of landmarks was repeated at a one-month interval by the same operator to assess intraoperator repeatability. The data were then imported into Excel spreadsheets (Microsoft Excel, Microsoft, Redmond, WA, USA) for the dental and statistical analyses.

### Dental analysis

A custom analysis to measure linear distances and angles incorporated a customized Excel file. In here, the scanner allocated a reference system to the digitized points randomly; it was necessary to re-establish a reference system related to the virtual dental cast. The new reference plane for both maxillary and mandibular dental casts was calculated as the plane passing through the intersection of the lingual developmental groove of the first permanent molar with the gingival margin (gingival limits of the lingual FACCs of the molars) and the calculated centroid of the gingival limits of the lingual FACCs of all the teeth (excluding ectopic canines when that condition occurred).

**Figure 2:**
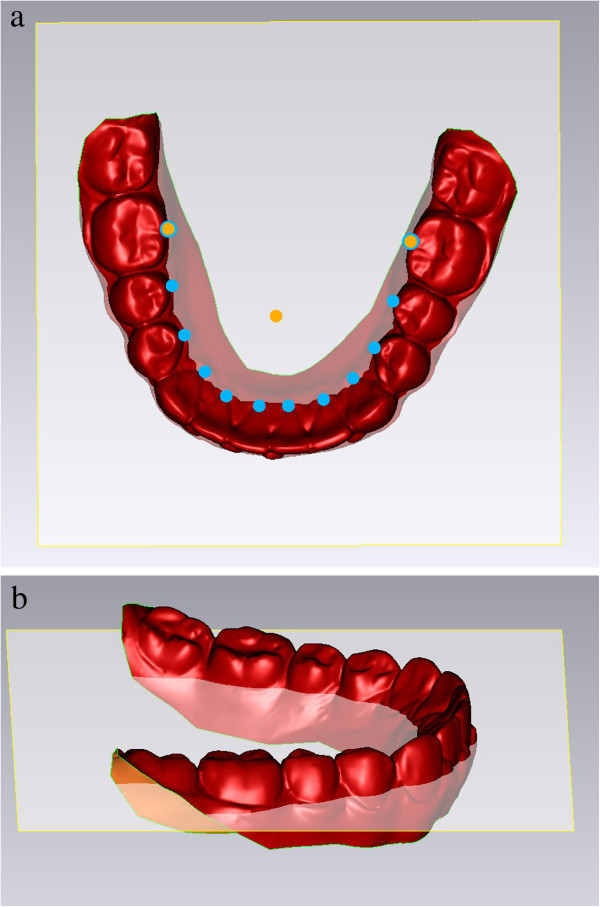
**An occlusal view (a) and a lateral view (b) of reference plane.**

The reference plane can be described as a best-fit plane among all of the lingual points, with the intermolar lingual distance set as the reference *X*-axis. This reference plane was constructed nearly parallel to the occlusal plane, avoiding variability due to tooth position and torque and curve of Spee, or curve of Wilson (Figure 2). The *X*-axis represented the transverse dimension, the *Y*-axis represented the sagittal dimension, and the *Z*-axis (perpendicular to the XY plane) represented the vertical dimension. All points were converted to the new reference plane through a three-dimensional rotational matrix.

Linear measurements were performed at this stage, while angular measurements required further computation.

### Angular measurements

*Torque* was measured as the labiolingual inclination of the FACCs and *tip* as the mesiodistal inclination of the FACCs relative to the reference plane. An individual tooth coordinate system, which follows each tooth, was necessary to determine such values. The mesial and distal points of each tooth were used for a second rotation of the XY plane, which determined the custom coordinate system for each tooth. The angles of torque and tip were then calculated using trigonometry. Lastly, a positive or negative sign was associated to the angle according to the same convention used for the bracket prescription (torque positive to the buccal and negative to the lingual, tip positive to the mesial and negative to the distal).

### Linear measurements

The linear distance measures included buccal, lingual, and centroid transverse widths at the level of canines, premolars, and molars as well as arch depth and arch perimeter. Three different transverse dimensions were measured for each pair of homologous teeth from canines to first molars: the transverse vestibular distance (TV), the transverse lingual distance (TL), and the transverse bodily distance (TB). The TV was calculated as the distance between the occlusal limits of the buccal FACCs of homologous teeth. The TL was calculated as the distance between the gingival limits of the lingual FACCs of the homologous teeth. The TB was calculated as the distance between the three-dimensional centroids of the homologous teeth.

To determine the centroid of the canines, premolars, and first molars, the midpoints of two lines passing from the mesial and distal landmarks (MD) and the gingival buccal and lingual limits (BL) of the FACCs were calculated. The midpoint of a line passing through these previously determined midpoints (MD and BL) was then determined. It was assumed that the centroid was the ‘center of mass’ of the clinical crown.

Arch depth was determined by measuring the length of a perpendicular line constructed from the mesial contact point of the central incisors to a line connecting the mesial points of the first molars [21]. The mesial contact point of the central incisors was calculated as the midpoint between the mesial points of the central incisors.

Arch perimeter was calculated as the sum (on the XY plane) of six segments (three per quadrant) extending from the mesial point of first molars to the mesial point of first premolars, from the mesial point of the first premolars to the distal point of lateral incisors, and from the distal point of lateral incisors to the mesial contact point of the central incisors. Arch depth and arch perimeter were calculated as a projection of the defined segments on the horizontal plane (XY plane), as defined in the literature [13, 21]. The set of calculated measures is shown in Table 1.

**Table 1:** **Statistics for the maxillary dentition**

Measure		***T*** test	ICC	MME	RME (%)
Torque	11	0.26	0.98	0.9	1.0
	12	0.56	0.98	1.2	1.3
	13	0.92	0.98	1.7	1.9
	14	0.18	0.94	1.6	2.1
	15	0.34	0.96	1.5	2.1
	16	0.98	0.87	1.7	2.3
	21	0.64	0.98	1.1	1.1
	22	0.88	0.98	2.2	2.4
	23	0.54	0.97	1.3	1.5
	24	0.72	0.96	2.2	2.9
	25	0.80	0.96	1.4	1.9
	26	0.86	0.92	1.5	2.1
Tip	11	0.90	0.92	2.0	2.1
	12	0.49	0.94	1.4	1.5
	13	0.12	0.90	1.5	1.6
	14	0.96	0.84	1.6	1.7
	15	0.45	0.81	1.8	2.0
	16	0.05	0.90	1.5	1.6
	21	0.89	0.89	1.6	1.7
	22	0.07	0.93	1.3	1.3
	23	0.14	0.94	2.6	2.7
	24	0.51	0.92	1.5	1.6
	25	0.41	0.78	2.2	2.4
	26	0.80	0.72	2.8	3.0
3 to 3	TV	0.97	0.98	0.3	0.7
	TL	0.37	0.98	0.5	1.9
	TB	0.67	0.97	0.2	0.7
4 to 4	TV	0.90	0.99	0.5	1.4
	TL	0.63	0.99	0.4	1.5
	TB	0.23	1.00	0.4	1.2
5 to 5	TV	0.47	0.99	0.5	1.2
	TL	0.84	0.99	0.3	1.0
	TB	0.19	1.00	0.3	0.9
6 to 6	TV	0.95	0.98	0.2	0.5
	TL	0.08	1.00	0.2	0.6
	TB	0.09	0.99	0.2	0.5
Arch depth		0.59	0.99	0.3	1.0
Arch perimeter		0.60	1.00	0.8	1.1

MME is the method error, and its values are in degrees for tip and torque and mm for all the other measurements. RME is the relative error magnitude.

### Statistical analysis

All dental casts for the 25 subjects were digitized twice by a single operator. The second digitization was repeated one month after the first digitization. Descriptive statistics were calculated for each linear and angular measurement at the two observation times. A normal distribution of data from both the first and second acquisitions was assessed through a Shapiro-Wilk test. A *t* test for paired samples (*p* < 0.05) was performed to assess the presence of systematic errors between the two observations.

Intraclass correlation coefficient with a two-way random effect model also was applied, checking for consistency between the two scores of the same rater. Intraclass correlation coefficient (ICC) values between 0.70 and 0.80 indicate a strong agreement while values greater than 0.80 indicate an almost perfect agreement between the two observations. To assess for repeatability and consistency of the dental cast analysis, the method error was calculated through the ‘method of moments’ estimator (MME) [22] and the relative error magnitude (REM) [23]. The mean and standard deviation of the random error for torque, tip, and linear measurements of the maxilla and of the mandible were calculated.

## Results

Tables 1 and 2 report the statistics relative to the systematic and random error for each angular and linear value of the maxilla and of the mandible, respectively. There was no systematic error; ICC values were higher than 0.70 on every measure.

**Table 2:** **Statistics for the mandibular dentition**

Measure		***T*** test	ICC	MME	RME (%)
Torque	31	0.24	0.98	0.8	0.9
	32	0.87	0.99	0.8	1.0
	33	0.96	0.97	1.2	1.7
	34	0.24	0.95	1.5	2.2
	35	0.84	0.98	1.2	2.1
	36	0.09	0.95	1.4	2.9
	41	0.17	0.98	0.9	1.0
	42	0.50	0.98	1.0	1.2
	43	0.45	0.96	1.2	1.6
	44	0.94	0.94	1.8	2.6
	45	0.29	0.98	1.1	1.8
	46	0.38	0.94	1.6	3.3
Tip	31	0.16	0.77	1.1	1.2
	32	0.51	0.88	1.5	1.6
	33	0.80	0.81	1.9	2.1
	34	0.44	0.89	1.7	1.9
	35	0.17	0.89	1.8	1.9
	36	0.48	0.70	3.5	3.7
	41	0.23	0.92	1.1	1.2
	42	0.05	0.87	1.6	1.9
	43	0.80	0.77	2.0	2.2
	44	0.17	0.84	1.8	1.9
	45	0.24	0.87	2.1	2.2
	46	0.57	0.74	3.8	4.0
3 to 3	TV	0.83	0.96	0.2	0.7
	TL	0.97	0.91	0.2	0.9
	TB	0.85	0.96	0.1	0.3
4 to 4	TV	0.67	0.98	0.2	0.6
	TL	0.74	0.99	0.1	0.4
	TB	0.58	0.99	0.1	0.2
5 to 5	TV	0.30	0.98	0.1	0.3
	TL	0.61	0.98	0.1	0.3
	TB	0.38	0.99	0.1	0.2
6 to 6	TV	0.09	0.98	0.2	0.5
	TL	0.68	0.99	0.1	0.3
	TB	0.79	0.98	0.1	0.2
Arch depth		0.13	0.98	0.1	0.4
Arch perimeter		0.07	0.99	0.2	0.2

MME is the method error, and its values are in degrees for tip and torque and mm for all the other measurements. RME is the relative error magnitude.

The average random error in the maxilla was 1.5° (±0.4°) for torque measures and 1.8° (±0.5°) for tip measures. The average random error for the linear measurements in the maxilla was 0.4 mm (±0.2 mm).

The average random error in the mandible was 1.2° (±0.3°) for the torque measures and 2.0° (±0.8°) for the tip measures. The average random error for the linear measurements in the mandible was 0.1 mm (±0.1 mm).

## Discussion

This study described and tested the reproducibility of a custom dental analysis performed on virtual three-dimensional study models. The shift from a standard ‘caliper and protractor’ analysis on plaster casts to a virtual three-dimensional analysis allows the introduction of new tools and measures in addition to the classic linear measures (transverse dimensions, arch depth, and arch perimeter).

The procedure proposed by Andrews [14] for measuring the FACCs inclinations was time consuming and required numerous steps for measuring the angulations and was potentially prone to error. According to the methodology proposed by Andrews, a ‘functional’ occlusal plane needed to be chosen, with the cast trimmed parallel to this occlusal plane. A protractor was used to measure the inclination of an axis tangent to a convex surface. This final step was the most controversial because the definition of a tangent to a convex, irregular surface might lead to inaccurate measures that are often difficult to replicate.

Using a similar methodology, Richmond and co-workers reported the range of error for the torque of the maxillary central incisors as 1.9° to 3.6° [16]. With the custom 3D dental analysis presented in the current study, we found a method error that ranged from 1.0° to 2.0° for the same teeth. The average method errors of the torque values for all teeth were 1.2° and 1.5° for the mandible and the maxilla, respectively, while the errors of tip values were 2.0° and 1.8° for the mandible and the maxilla, respectively.

Ferrario and co-workers, using a methodology similar to that reported in the current study, also incorporated the use of an electromagnetic digitizer. These investigators reported a method error of 2.5° and 2.3° on the sagittal and frontal planes, respectively [18]. The linear measure error reported by Ferrario and co-workers [18] was 0.2 mm (calculated for the crown height length) while average method of errors of 0.1 and 0.2 mm for the mandibular and maxillary linear measures, respectively, were reported in the current study.

The relative error magnitude in the present study ranged from 0.9% to 4.0% for the angular measures and 0.1% to 1.9% for the linear measures. Both the method error and the relative error magnitude indicate a good degree of reproducibility of both the linear and angular measures. The additional but necessary step of setting a custom reference system to calculate tip and torque angles may account for the higher degree of variation of the angular measures when compared to the linear measures. Also, the error increases as the number of landmarks necessary for the measurement increases, as reported by Luu and co-workers [24].

The definition of the tip and torque values as the actual inclination of a segment passing through the gingival and occlusal limits of the FACC may account for an improved reproducibility compared to manual measures with a protractor, as described previously in the literature [14–17]. The errors of the proposed method may be larger in longitudinal studies for comparisons of before- and after-treatment changes or in any clinical situation that potentially changes the clinical crown, both in the occlusogingival and the buccolingual dimensions. Examples include attrition of the occlusal surface due to bruxism, poor restorations, gingival inflammation, severe rotations, intrusion/extrusion biomechanics, and teeth that are not fully erupted due to an early stage of maturation or a lack of space. The relative change of the gingival or occlusal limit of the FACC may account for an error in the estimation of the tooth inclination with respect to the reference plane.

The validation of the digital dental analysis in this study allows for the measurement of tip and torque and potentially can be applied to better understand the nuances of different bracket prescriptions. This new tool may be useful to both the clinician and the researcher as it may allow a better understanding of the changes that occur due to growth or to treatment when comparing dental casts at two different time points. Three-dimensional virtual dental cast analysis may be encouraged, as it provides additional information and more accurate intra-arch measurements than traditional stone cast analysis.

## Conclusions

A custom dental analysis to measure traditional linear measurements of virtual dental casts as well as tip and torque angulation of individual teeth was presented. This validation study demonstrated that the virtual dental cast analysis presented in this report has adequate reproducibility, providing additional information and more accurate intra-arch measurements for clinical diagnosis and dentofacial research.
